# Association of a Homozygous *TYMP* c.131G>C Variant With MNGIE in a Chinese Pedigree: Insights From Genetic Analysis and Computational Modeling

**DOI:** 10.1002/mgg3.70253

**Published:** 2026-06-17

**Authors:** Ling Li, Xiu Chen, Hua Li, Kai Guo, Ting Zhang, Mingyi Ma, Songhua Zhao

**Affiliations:** ^1^ Department of Neurology Affiliated Hospital of Southwest Medical University Luzhou China; ^2^ Department of Endocrinology Affiliated Hospital of Southwest Medical University Luzhou China; ^3^ Department of Medical Cell Biology and Genetics, School of Basic Medical Sciences Southwest Medical University Luzhou China

**Keywords:** missense variant, MNGIE, nucleotide homeostasis, *TYMP*, whole‐exome sequencing

## Abstract

**Background:**

Mitochondrial neurogastrointestinal encephalomyopathy (MNGIE) is an autosomal recessive disorder caused by mutations in *TYMP*, which disrupt thymidine metabolism. This study aimed to characterize a novel homozygous *TYMP* variant and provide insights into its potential structural and functional consequences through bioinformatic analyses.

**Methods:**

We identified a homozygous *TYMP* variant (c.131G>C, p.R44P) in a proband with MNGIE using whole‐exome sequencing and Sanger sequencing. Computational structural analyses and molecular modeling were performed to predict the impact of the R44P substitution on thymidine phosphorylase (TP) stability, homodimerization, catalytic activity, and substrate binding.

**Results:**

The homozygous *TYMP* c.131G>C variant was confirmed in the proband. Computational analyses suggested that the p.R44P substitution may destabilize TP and potentially impair homodimerization. Molecular modeling further predicted altered thymidine binding and disrupted active‐site geometry. These predicted perturbations are hypothesized to contribute to defective nucleotide metabolism, thymidine accumulation, and deoxynucleotide triphosphate pool imbalance, which may ultimately result in mitochondrial genomic instability manifesting as mitochondrial DNA deletions and depletion.

**Conclusion:**

Our findings report the *TYMP* c.131G>C variant in a homozygous configuration, extending beyond a recently described compound heterozygous case. The bioinformatic predictions support the classification of this variant as likely pathogenic in MNGIE, though functional studies are warranted to validate these findings.

## Graphical abstract

Researcher highlight: Extending beyond the recently reported compound heterozygous case, we report the same *TYMP* c.131G>C variant in a homozygous configuration, delivering key genetic evidence for its standalone pathogenicity in MNGIE.

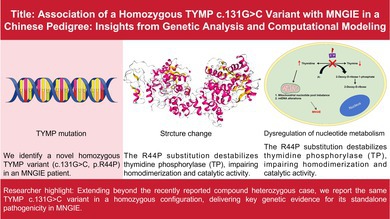

## 1 Introduction

Mitochondrial neurogastrointestinal encephalomyopathy (MNGIE) (OMIM: 603041) is a rare autosomal recessive disorder caused by biallelic mutations in the Thymidine Phosphorylase Gene (*TYMP*), which encodes Thymidine Phosphorylase protein (TP protein) (Nishino et al. 1999). Clinically, MNGIE manifests between the first and fifth decades of life, featuring a multisystemic phenotype including extraocular muscle weakness, severe gastrointestinal dysmotility, cachexia, peripheral neuropathy, leukoencephalopathy, and sensorineural hearing loss, characterized by secondary mtDNA deletions, depletion, and point mutations resulting from nucleotide pool imbalance (Nalini and Gayathri 2011; Nishino et al. 2001). With an estimated prevalence of 1–9 per 1,000,000 individuals (Khan et al. 2022), MNGIE illustrates the critical role of nucleo‐mitochondrial crosstalk in maintaining mtDNA integrity, as TP dysfunction disrupts thymidine homeostasis, leading to toxic accumulation of thymidine and deoxyuridine in plasma and tissues (Laforce et al. 2009).

TP, a cytosolic enzyme that phosphorylates thymidine and deoxyuridine (Felhi et al. 2019), is broadly expressed in macrophage‐like cells, placental tissue, and immune organs (e.g., spleen, lymph nodes), with detectable activity in the liver, lungs, and peripheral lymphocytes. Paradoxically, *TYMP* overexpression is implicated in tumor progression across diverse cancers—including carcinomas of the breast, lung, gastrointestinal tract, and reproductive systems (Sifeddine et al. 2024)—highlighting its context‐dependent roles in metabolism and disease.

In the current study, we characterize a Chinese pedigree with MNGIE syndrome that harbors a homozygous *TYMP* missense variant (c.131G>C, p.R44P). Whole‐exome sequencing, validated by Sanger sequencing, revealed complete segregation of this variant with the disease phenotype. Computational structural analyses provided supporting evidence for the likely pathogenicity of this *TYMP* variant and showed its effects on TP structure, stability and activity.

## 2 Patient and Methods

### 2.1 Editorial Policies and Ethical Considerations

Informed consent was obtained from all participating subjects. The research protocol was conducted in accordance with the Declaration of Helsinki guidelines and approved by the Ethics Committee of the Affiliated hospital of Southwest Medical University (Approval No. KY2023410).

### 2.2 Clinical Description of the Proband

A 36‐year‐old man presented to the Department of Neurology, Affiliated Hospital of Southwest Medical University, with a 6‐month history of progressive dizziness, tinnitus, and hearing impairment.

At symptom onset (approximately 6 months prior to admission), the proband reported experiencing dizziness and tinnitus accompanied by hearing impairment without an obvious cause. The symptoms were not associated with vertigo, altered consciousness, limb numbness or weakness, gaze fixation, nausea, vomiting, coughing, palpitations, chest tightness, chest pain, abdominal pain, diarrhea, or other discomforts. Initially, the proband did not seek medical attention; however, as the symptoms progressively worsened, he eventually presented to our department for evaluation.

Upon admission (at age 36), the proband exhibited a thin build (BMI: 16.6 kg/m^2^) with marked muscle atrophy in the face, trunk, and limbs, along with pes cavus and deformities of both feet and knee joints. According to the proband, this low body weight had been stable, with no significant weight change reported over the preceding 6 months. He was alert but had blurred vision and mildly slurred speech. No ptosis was observed in either eye. Baseline laboratory investigations, including complete blood count (CBC), liver function tests (LFTs), and renal function tests (RFTs), were performed. The results of these and other routine clinical laboratory tests are provided in the Supporting Information. Routine stool examination and pre‐transfusion testing revealed no significant abnormalities. A remote electrocardiogram (ECG) showed sinus rhythm with a fragmented QRS complex in lead V1. Echocardiography revealed mild tricuspid regurgitation, normal left ventricular systolic function, and a minimal pericardial effusion.

The proband's presentation raises suspicion for Charcot–Marie–Tooth disease, other inherited disorders, or malignancy. Following discussion among the neurology team, whole‐exome sequencing (WES) was initiated to assess potential genetic defects.

### 2.3 Gene Sequencing

Blood samples were collected from the proband (II‐2), his unaffected parent (I‐2), and sister (II‐3). Whole‐exome sequencing of the proband was performed at MyGenostics Sequencing Service (Beijing, China). The whole exomes in affected individuals, along with adjacent intron regions (50 bp), were captured via the GenCap whole‐exome capture kit, amplified, and high‐throughput sequenced using the DNBSEQ‐T7 to prepare the DNA library. Then the sequencing data were mapped to the UCSC hg19 human reference genome through the parameter BWA in the Sentieon software for variation detection. Sanger sequencing was also conducted in this pedigree to verify their variation results and whether other unaffected individuals carried the same suspected variant.

### 2.4 Protein Domain Analysis

Functional domains and critical sites of the protein were predicted using InterPro (EMBL‐EBI), a comprehensive tool for protein sequence analysis and classification. This resource integrates predictive signatures from multiple databases to provide robust domain architecture annotations. We employed InterPro to map the positions of nonsynonymous single nucleotide polymorphisms (nsSNPs) within identified protein domains, enabling structural and functional interpretation of genetic variants.

### 2.5 Bioinformatics Analyses

Bioinformatics analyses were conducted by mapping the sequencing data to multiple databases, including OMIM, Clinvar, dbSNP, and HGMD. Single nucleotide variants (SNVs) were identified using the parameter driver of the Sentieon software. Based on the Standards and Guidelines for the Interpretation of Sequence Variants of the American College of Medical Genetics and Genomics (ACMG), variation pathogenicity was assessed. SIFT, MutationTaster, GERP+, and REVEL were also employed to predict the variation pathogenicity. Clustal Omega was utilized for the conservation analyses of the variant sites across different species. In addition, I‐TASSER (J. Yang and Zhang 2015; Zheng et al. 2021; Zhou et al. 2022) was applied to predict the spatial conformation of the protein, changes in binding ligand types, binding sites, enzyme commission (EC) number, and active sites. Pymol was used to analyze the 3D structural difference between the wild‐type and mutant protein.

### 2.6 Molecular Docking Analysis

Protein‐ligand interactions were analyzed using CB‐Dock2 (Liu et al. 2022; X. Yang et al. 2022), an automated docking platform for cavity detection and binding pose prediction. We performed comparative docking studies of thymine with both wild‐type (WT) and mutant TP enzyme structures to evaluate mutation‐induced alterations in binding energetics and interaction patterns. Binding conformations were assessed based on calculated binding affinities and detailed analysis of active site interactions in both monomeric and homodimeric forms.

## 3 Results

### 3.1 Patient Report and Radiodiagnosis

**FIGURE 1:**
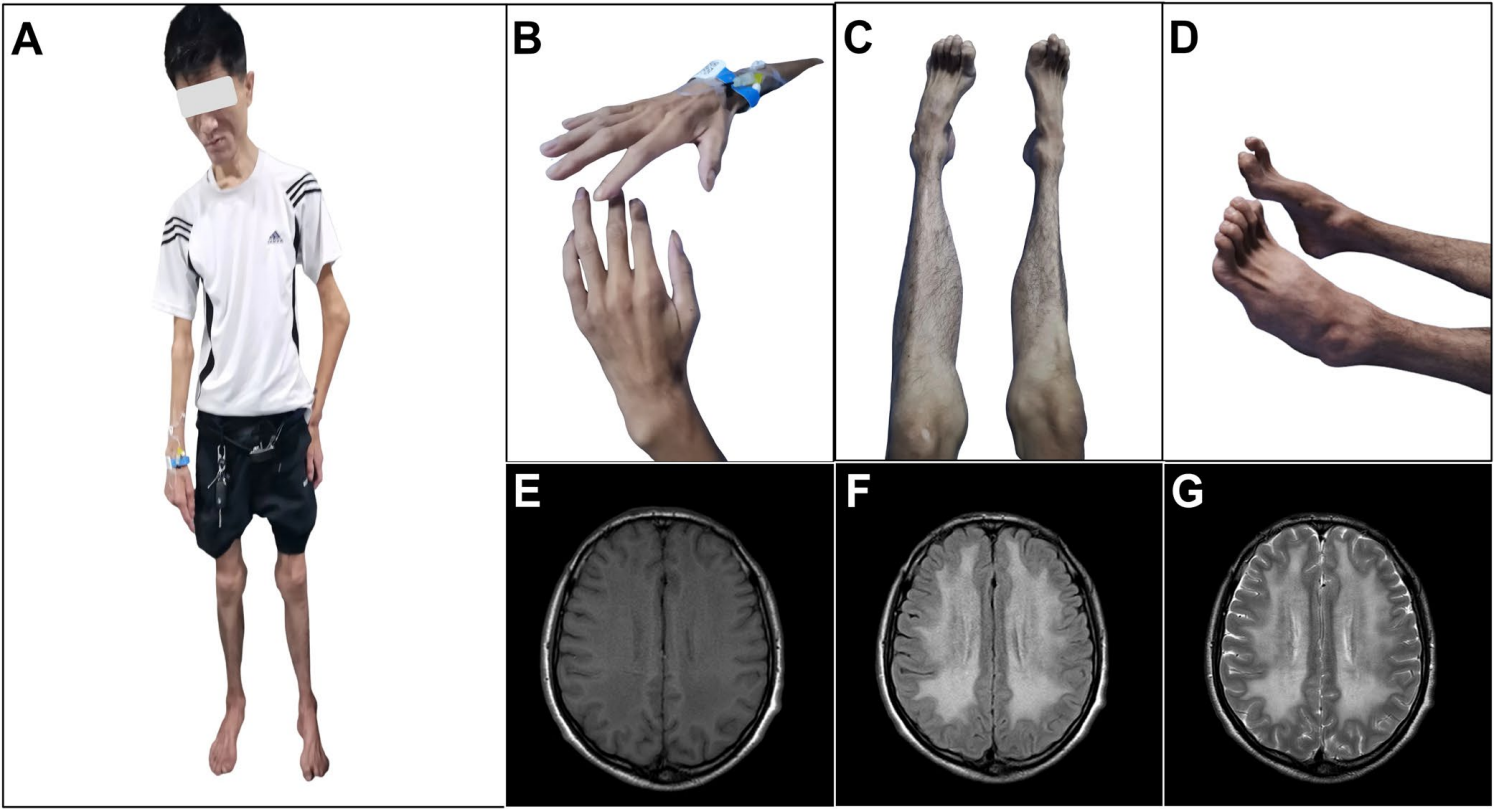
Clinical features in the proband. (A–D) Whole body picture of the proband showed a thin build with marked muscle atrophy in the face, trunk, and limbs, along with pes cavus and deformities of both feet and knee joints. (E‐G) Brain MRI performed in the proband revealed bilateral white matter abnormalities, showing T1 hypointensities (E) with corresponding T2‐FLAIR (F) and T2WI (G) hyperintensities.

The proband exhibited a thin build with marked muscle atrophy in the face, trunk, and limbs, along with pes cavus and deformities of both feet and knee joints (Figure 1A–D).

Brain Magnetic resonance imaging (MRI) in the proband revealed bilateral white matter abnormalities, showing T1 hypointensities (Figure 1E) with corresponding T2‐FLAIR (Figure 1F) and T2WI (Figure 1G) hyperintensities.

### 3.2 Whole‐Exome and Sanger Sequencing

**FIGURE 2:**
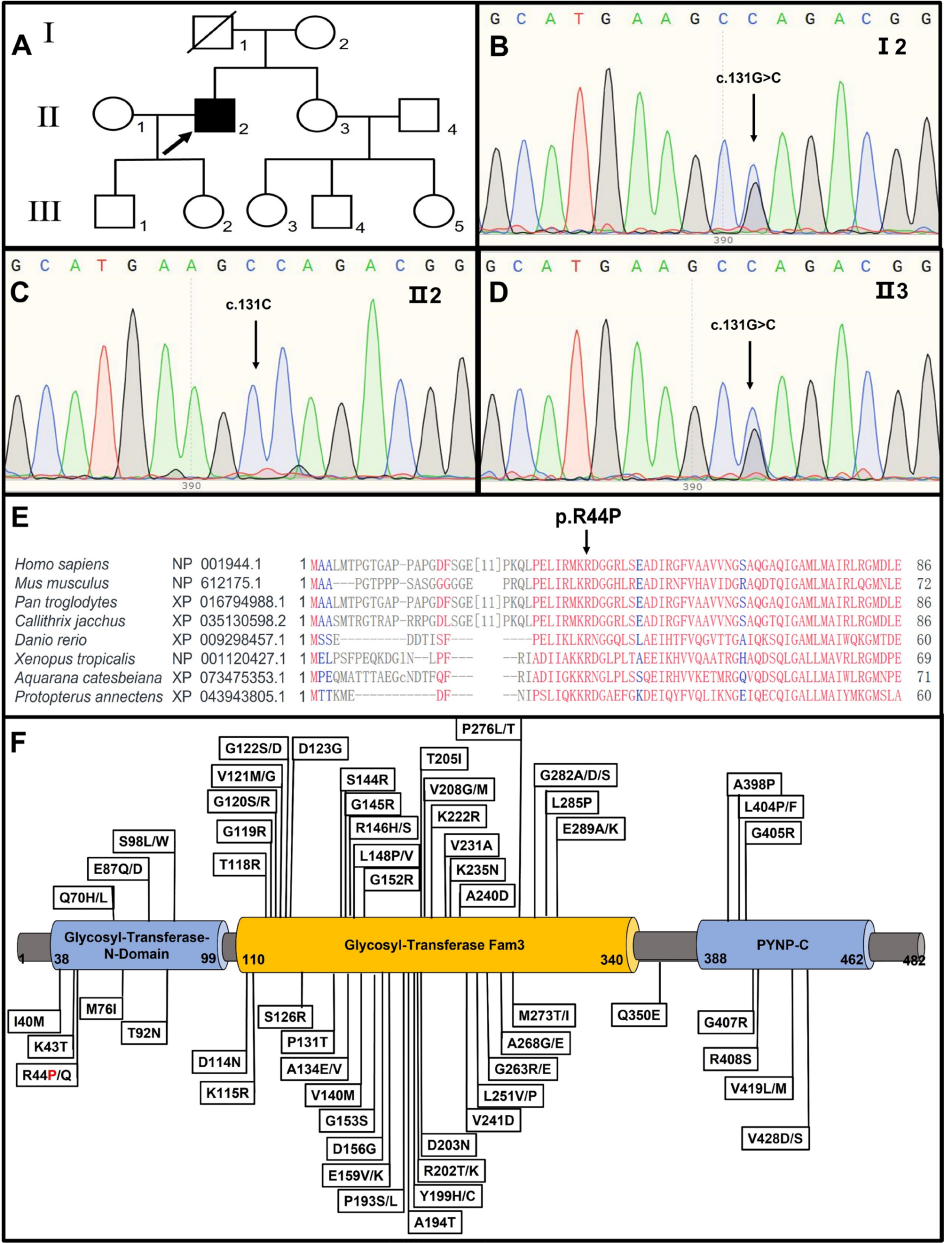
Pedigree structure of this MNGIE family and Sanger sequencing validation of *TYMP* c.131G>C and conversational analyses of TP locus 44 aa across different species and location of different pathogenic variants in the human TP protein. The red P in panel E means the novel pathogenic variant found in our report. The panel F was modified from (Sifeddine et al. 2024).

Among the sequencing results, high‐frequency polymorphisms and variants unrelated to nucleotide salvage pathway were excluded. We identified a homozygous missense c.131G>C (p.R44P) variant in *TYMP* (NM_001953.5; exon 2) in II2, causing an amino acid substitution from R to P at position aa‐44. Subsequent Sanger sequencing confirmed this variant in affected individuals I2 and II3 (Figure 2A–D).

### 3.3 Variant Characterization and ACMG Classification

The identified homozygous *TYMP* variant (c.131G>C, p.R44P) was absent in the gnomAD database (v4.1.0), indicating extreme rarity in the general population. Cross‐species alignment using Clustal Omega revealed absolute conservation of arginine at position 44 across eight vertebrate species (Figure 2E), suggesting functional importance of this residue.

Domain analysis using InterPro localized the p.R44 residue within the N‐terminal Glycosyl‐Transferase‐N domain (residues 38–99), one of three functionally critical regions of the TP protein (Figure 2F). Notably, mapping of non‐synonymous SNPs showed significant clustering of evolutionarily conserved variants within these domains, further supporting their functional relevance.

Based on ACMG guidelines, the variant was classified as likely pathogenic (Class 4), meeting the following criteria: PM2 (absent from gnomAD), PM3 (same residue position previously implicated in MNGIE, R44Q), PP3 (multiple in silico tools predict damaging effect; REVEL score 0.718), and PP4 (patient phenotype highly specific for MNGIE). Parental segregation data were unavailable, precluding a definitive pathogenic classification.

### 3.4 Predicted Structural Impact of the p.R44P Variant

**FIGURE 3:**
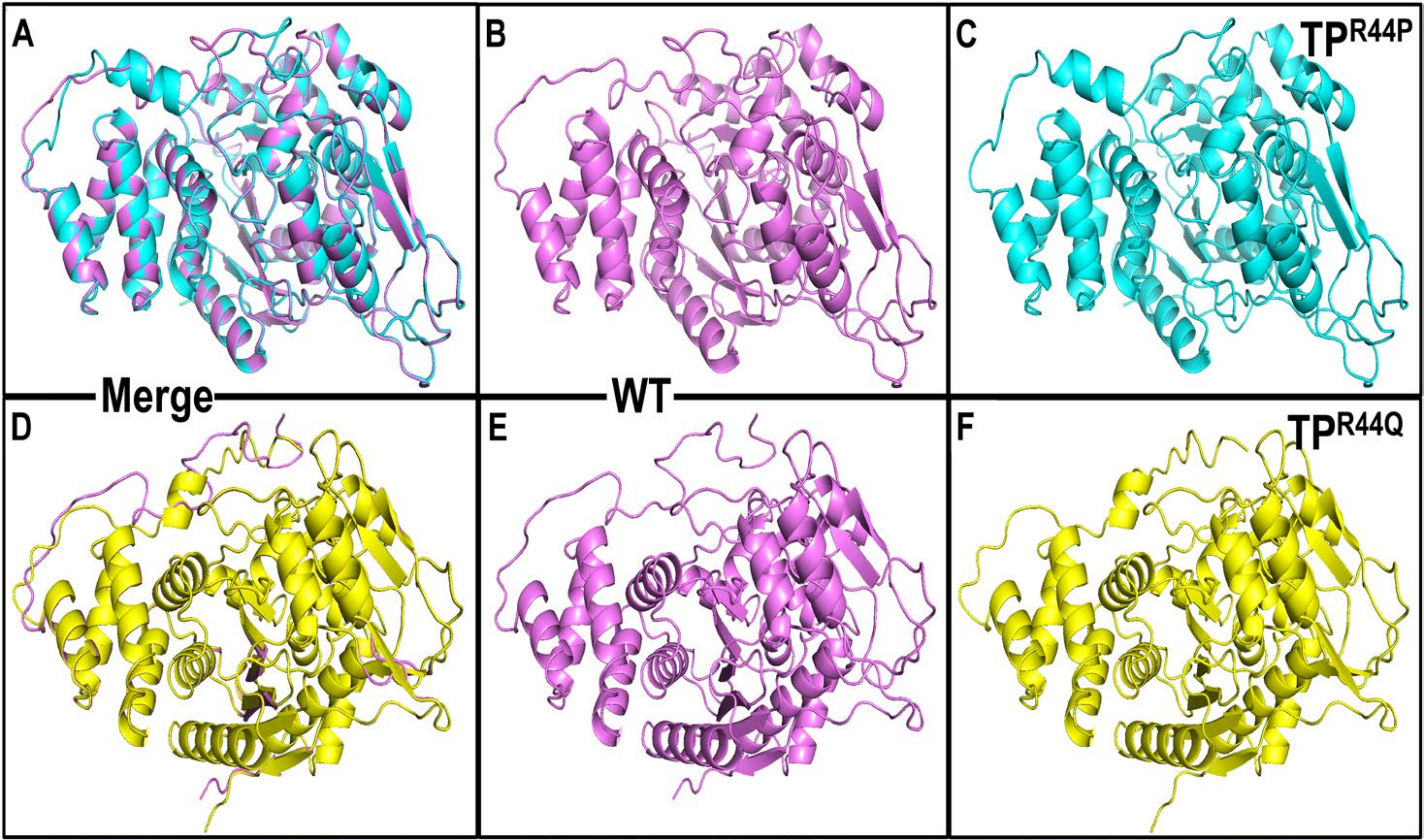
Comparative structural modeling prediction of wild‐type TP, TP^R44P^ and TP^R44Q^ variant. (A–C) Structural analysis identified 12 amino acid substitutions distributed across six discrete domains of the pathogenic variant protein. (D, E) Structural characterization of the pathogenic variant protein revealed six distinct clusters of amino acid substitutions.

To explore the potential consequences of the p.R44P substitution, we performed comparative protein structure modeling using I‐TASSER. Superimposition of wild‐type and mutant TP models revealed predicted conformational changes localized to the N‐terminal domain (Figure 3A–E).

Given that a distinct substitution at the same residue (R44Q) has been previously reported in MNGIE (Gamez et al. 2002), we extended our analysis to compare the predicted effects of both variants. Secondary structure predictions indicated that: TP‐R44P was associated with helix extension and β‐sheet modifications (Table 1), and TP‐R44Q was associated with gain of secondary structures and β‐sheet truncations (Table 2).

**TABLE 1:** Detailed residue‐level comparison of predicted secondary structure changes in TP‐R44P.

Structural change	Wild‐type	TP‐R44P
Gain of α‐helix	Loop/coil	15–22 aa (New α‐helix)
Elongated α‐helices	129–135 aa, 213–224 aa, 354–359 aa, 394–403 aa	129–136 aa, 211–224 aa, 354–361 aa, 394–404 aa
Shortened β‐sheets	112–117 aa, 139–144 aa, 229–236 aa, 266–271 aa	113–117 aa, 140–144 aa, 229–233 aa, 266–268 aa
Extended β‐sheets	379–382 aa, 437–440 aa	378–382 aa, 437–441 aa
Complete loss of β‐sheet	474–477 aa (β‐sheet)	Loop/coil

*Note:* The most prominent predicted changes include the gain of a novel α‐helix (residues 15–22) and the complete loss of a C‐terminal β‐sheet (residues 474–477), suggesting potential impacts on protein folding and stability. Other changes involve modest shifts in the boundaries of existing helices and sheets.

**TABLE 2:** Detailed residue‐level comparison of predicted secondary structure changes in TP‐R44Q.

Structural change	Wild‐type	TP‐R44Q
Gain of α‐helix	Loop/coil	16–21 aa (New α‐helix)
De novo β‐sheets	Loop/coil	388–391 aa, 459–462 aa
β‐sheet truncations	112–117 aa, 139–144 aa, 229–236 aa	114–117 aa, 140–144 aa, 230–235 aa

*Note:* The most prominent predicted changes include the gain of an N‐terminal α‐helix (16–21) and de novo formation of two β‐sheets (388–391, 459–462), features that distinguish R44Q from the R44P mutant (see Table 1).

### 3.5 Molecular Docking Simulations of Thymine Binding

**FIGURE 4:**
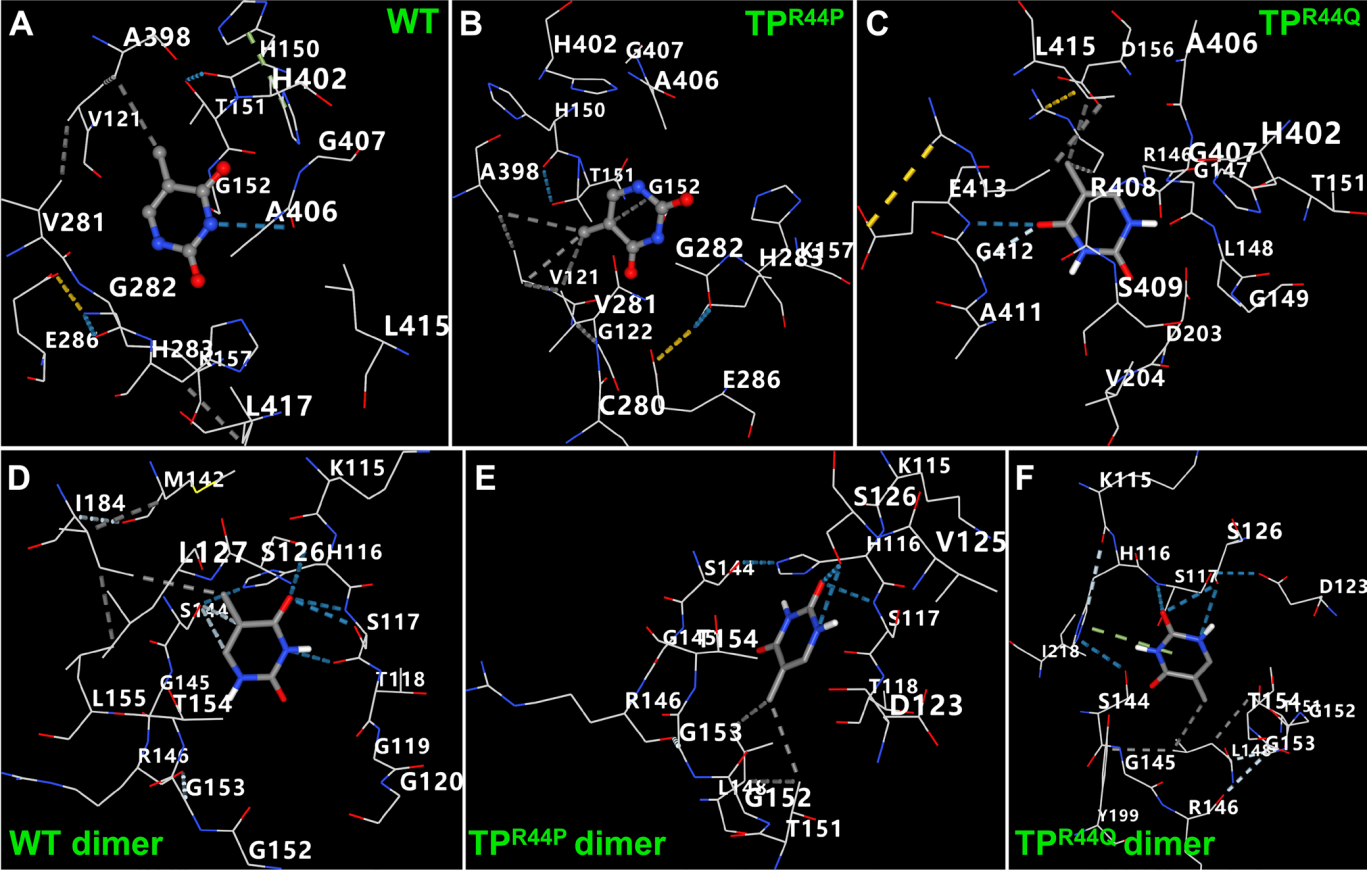
Molecular docking of TP and Thymine. (A) Thymine's methyl group shows proximity to F398's phenyl ring. Its N3 hydrogen bonds with F398's backbone carbonyl oxygen. (B) Thymine's methyl group interacts with F398, V121, and R44P side chains. No hydrogen bonds are observed at this interface. (C) Thymine's methyl group contacts L415, while its O4 carbonyl forms a hydrogen bond with the G412‐E413 peptide backbone. (D) thymine's methyl group interacts with F398, while its methylene groups position near S144; the nucleobase forms an extensive hydrogen‐bond network involving O2 (with S117, S126, and H116‐S117 backbone) and N3 (with S117 hydroxyl and T118 backbone amide). (E) The TPR44P dimer binds thymine through methyl group contacts with L148/T151 side chains, complemented by hydrogen bonds between thymine's O4 and S126 hydroxyl plus H116‐S117 backbone, and between N3 and S126 hydroxyl. (F) Thymine engages the TPR44Q dimer via methyl group interaction with L148, while establishing hydrogen bonds through N3 (with S126 hydroxyl) and O4 (with S126 hydroxyl and H116‐S117 backbone).

To predict how the p.R44P and p.R44Q substitutions might affect ligand binding, we performed molecular docking simulations of thymine with wild‐type and mutant TP proteins (Figure 4A–F). These results represent computational predictions and should be interpreted as suggestive of possible functional effects, rather than definitive evidence.

#### 3.5.1 Monomer Interactions

In the wild‐type TP monomer (Figure 4A), thymine's methyl group positioned near Phe398, with a hydrogen bond between its secondary amine and Phe398's carbonyl oxygen. The TP‐R44P mutant (Figure 4B) exhibited predicted proximity between thymine's methyl group and Phe398, Val121, and the mutant side chain. For TP‐R44Q (Figure 4C), thymine's methyl group neighbored Leu415, with its carbonyl oxygen hydrogen‐bonding to Gly412‐Glu413.

#### 3.5.2 Dimer Interactions

Dimer configurations showed more extensive predicted interactions. Wild‐type dimers (Figure 4D) positioned thymine near Phe398 and Ser144, with multiple hydrogen bonds to Ser117/126, Thr118, and His116. Both TP‐R44P (Figure 4E) and TP‐R44Q (Figure 4F) dimers localized thymine near Leu148/Thr151, with hydrogen bonds to Ser126 and His116/Ser117. While the overall binding patterns were similar between the two mutants, subtle differences in steric constraints were observed.

### 3.6 Summary of Reported Codon 44 Variants in *TYMP*

To provide a comprehensive overview of variants affecting this critical residue, we summarized all previously reported codon 44 variants in the *TYMP* gene, along with the variant identified in this study (Table 3). This summary highlights the diversity of genetic alterations at this position and their associated clinical phenotypes.

**TABLE 3:** Novel and previously reported codon 44 variants in *TYMP* with clinical phenotypes.

Amino acid change	Nucleotide change	First reported	Population	TP enzyme activity	Key clinical features	Classification
R44Q	c.131G>A	(Gamez et al. 2002)	Spanish	Reduced	GI dysmotility, extraocular muscle weakness, phenotypic variability	Pathogenic
R44X	c.130C>T	(Mojtabavi et al. 2021)	Iranian	Not available	Severe cachexia, ptosis, diplopia, hearing impairment, abdominal cramps	Pathogenic
R44Q	Not applicable (computational)	(Bhagat et al. 2025)	N/A	Not available	Not applicable (computational study)	N/A
R44P (Our study)	c.131G>C	2025	Chinese	Not available	Thin build, muscle atrophy, pes cavus, joint deformities, no ptosis, no GI symptoms	Likely pathogenic
R44P	Compound heterozygous (c.131G>C/c.1268 T>G)	(Xu et al. 2025)	Chinese	Not available	GI dysmotility, leukoencephalopathy, peripheral neuropathy	Likely pathogenic

## 4 Discussion

MNGIE is a rare autosomal recessive disorder caused by mutations in the *TYMP* gene, which encodes TP protein, a critical enzyme in pyrimidine homeostasis. In this study, we report a Chinese proband with a homozygous *TYMP* variant (c.131G>C, p.R44P). We describe the clinical presentation, characterize the variant, and use computational modeling to predict its structural consequences. Our findings expand the variational spectrum of *TYMP* and highlight the clinical heterogeneity of MNGIE.

### 4.1 Clinical Heterogeneity: Beyond the Classic Phenotype

The phenotypic spectrum of MNGIE has expanded considerably beyond the classic tetrad of gastrointestinal dysmotility, cachexia, ptosis/ophthalmoplegia, and peripheral neuropathy. A comprehensive position paper from the MNGIE International Network has highlighted the expanding recognition of phenotypic variability and the importance of considering atypical presentations in the diagnostic pathway (Hirano et al. 2021).

Our proband exhibited several features that underscore this clinical heterogeneity. Notably, he presented with prominent musculoskeletal deformities—including pes cavus and bilateral foot and knee joint abnormalities—in the absence of ptosis and gastrointestinal symptoms, two hallmark features of classic MNGIE. His initial symptoms were dominated by dizziness, tinnitus, and hearing impairment, which are less commonly emphasized in classic descriptions. These atypical presentations align with a growing body of evidence that *TYMP*‐related disease can manifest beyond the classic gastrointestinal and neurological tetrad.

Recent reports have further broadened the recognized spectrum. Notably, a case report documented growth hormone deficiency as an endocrine manifestation of MNGIE (Sawafta et al. 2025), illustrating the multisystem nature of this disorder. As Garone et al. demonstrated in a landmark cohort analysis, even patients carrying the same *TYMP* mutation can exhibit marked differences in age of onset, symptom combination, and disease severity, indicating that clear genotype–phenotype correlations are not straightforward (Garone et al. 2011). Together, these observations suggest that MNGIE may be conceptualized as a multisystem mitochondrial disorder with variable expressivity, rather than a narrowly defined syndromic entity. Increased awareness of this clinical heterogeneity is essential for timely diagnosis, particularly in patients presenting with atypical or incomplete phenotypes.

### 4.2 Molecular Mechanisms: From Gene to Mitochondrial Dysfunction

How do *TYMP* mutations lead to such diverse clinical presentations? The answer may lie in the central role of TP in nucleotide metabolism. TP functions as a homodimeric enzyme that catalyzes the reversible phosphorolysis of thymidine and deoxyuridine into their corresponding bases and 2‐deoxyribose‐1‐phosphate (Bhagat et al. 2025). This reaction is critical for maintaining thymidine homeostasis.

When TP function is impaired, toxic levels of thymidine and deoxyuridine accumulate. These excess nucleosides may disrupt mitochondrial DNA (mtDNA) replication fidelity, leading to progressive acquisition of secondary mtDNA mutations and, eventually, mtDNA depletion (Gautheron et al. 2022). This molecular cascade compromises oxidative phosphorylation, creating a cellular energy crisis that manifests clinically as multi‐organ dysfunction. Notably, different mutations in *TYMP* can result in partial or complete loss of TP activity, which may explain, at least in part, the clinical heterogeneity observed in MNGIE patients (Mojtabavi et al. 2021).

### 4.3 Comparison With Previously Reported Codon 44 Variants

The arginine residue at position 44 of the TP protein is highly conserved across species (Figure 2E) and located within the N‐terminal Glycosyl‐Transferase‐N domain, suggesting its functional importance. Gamez et al. first reported a mutation at this residue—R44Q (c.131G>A)—in a patient with classic MNGIE (Gamez et al. 2002). More recently, a large‐scale computational study by Bhagat et al. (Bhagat et al. 2025) systematically analyzed the structural and dynamic impacts of MNGIE‐associated mutations, including R44Q, suggesting that this substitution may induce substantial protein destabilization, increased flexibility, and reduced thymidine‐binding affinity.

In this study, we identified a distinct substitution at the same residue—R44P (c.131G>C). While both variants affect the same amino acid position and lead to the classic MNGIE phenotype, several observations merit discussion. First, the R44P substitution introduces a proline residue, which is known to disrupt protein secondary structure by potentially introducing a rigid kink in the polypeptide backbone. Second, our structural modeling predicted distinct alterations: R44P was associated with helix extension and β‐sheet modifications, whereas R44Q was predicted to induce gain of secondary structures and β‐sheet truncations (Tables 1 and 2). These distinct predicted structural alterations suggest that different molecular mechanisms may converge on the same clinical phenotype.

Clinically, both patients presented with the core features of MNGIE. However, our proband exhibited a notable absence of ptosis and gastrointestinal symptoms, along with prominent musculoskeletal deformities (pes cavus, bilateral foot and knee joint abnormalities), raising the possibility of subtle phenotypic differences between these two substitutions. Additionally, the initial presentation with dizziness, tinnitus, and hearing impairment—symptoms less commonly emphasized in classic MNGIE descriptions—may suggest a broader phenotypic spectrum associated with variants at this residue. Given the known phenotypic heterogeneity in MNGIE and the lack of clear genotype–phenotype correlations (Garone et al. 2011), definitive conclusions would require functional studies and additional case reports. Nevertheless, the identification of a second pathogenic substitution at codon 44 reinforces the critical role of this residue in TP function.

### 4.4 Structural Consequences: Distinct Perturbations Induced by R44P and R44Q

To explore how the p.R44P substitution might impair TP function, we performed comparative protein structure modeling using I‐TASSER. As summarized in Tables 1 and 2, the two variants were predicted to induce distinct secondary structure alterations. R44P was associated with helix extension, β‐sheet modifications, and a complete loss of a C‐terminal β‐sheet (residues 474–477). In contrast, R44Q was predicted to exhibit de novo β‐sheet formation (residues 388–391, 459–462) and truncation of existing β‐sheets. These distinct structural fingerprints suggest that different amino acid substitutions at the same residue may perturb TP function through divergent molecular mechanisms.

Our predictions are consistent with recent computational work by Bhagat et al. (Bhagat et al. 2025), who used molecular dynamics simulations to demonstrate that MNGIE‐associated *TYMP* mutations, including R44Q, induce substantial protein destabilization and increased flexibility. Our findings extend this work by providing a comparative structural analysis of two different substitutions at codon 44, highlighting the potential for divergent molecular mechanisms to converge on the same disease phenotype.

### 4.5 Functional Implications: Effects on Ligand Binding and Dimerization

**FIGURE 5:**
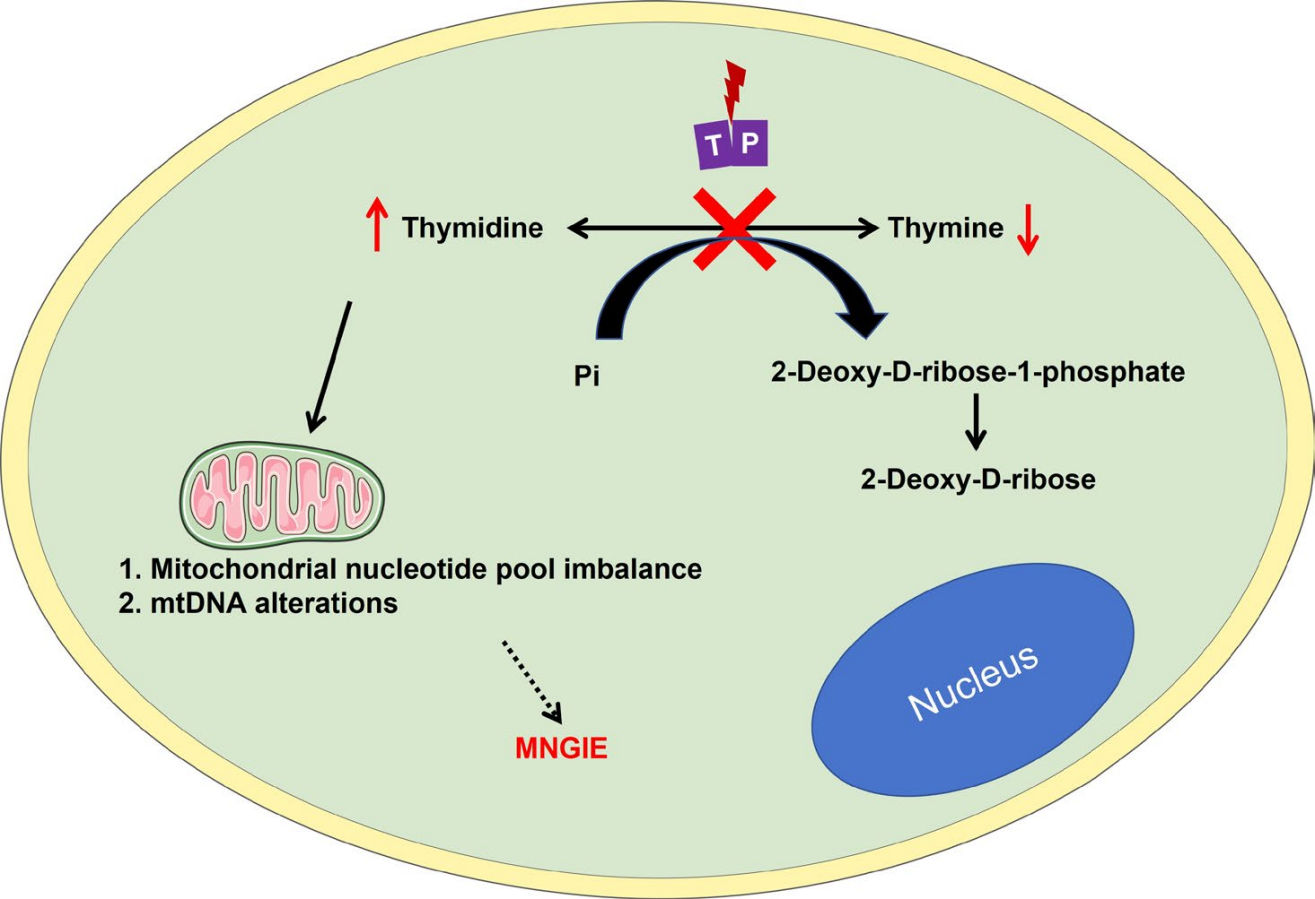
Schematic drawing showing the functional impact of *TYMP* variant. Thymidine phosphorylase (TP) mediates the reversible phosphorolytic cleavage of thymidine into thymine and 2‐deoxy‐D‐ribose‐1‐phosphate, the latter being subsequently metabolized to 2‐deoxy‐D‐ribose. Impaired TP function disrupts this catalytic process, leading to an accumulation of thymidine and a concomitant reduction in thymine levels. This metabolic imbalance may compromise mitochondrial genome stability by promoting mtDNA alterations and dysregulating the thymidine salvage pathway, ultimately contributing to the pathogenesis of MNGIE.

To assess whether these structural changes may affect protein function, we performed molecular docking simulations to predict how the R44P and R44Q substitutions might alter thymine binding (Figure 5).

Our simulations predicted distinct variant‐dependent alterations. Wild‐type TP was predicted to maintain conserved interactions with Phe398. In contrast, R44P was predicted to introduce new hydrophobic contacts with Val121 and the mutant side chain, while R44Q shifted binding orientation toward Leu415 and formed novel hydrogen bonds with Gly412‐Glu413. In the dimeric forms, both mutants exhibited altered thymine positioning—Leu148/Thr151 for R44P and Leu148 for R44Q—along with distinct steric constraints. These predicted perturbations likely impair homodimer formation, which is critical for TP enzymatic efficiency and stability (Bhagat et al. 2025). Collectively, these structural and docking analyses suggest that the R44P substitution compromises TP function, leading to the downstream mitochondrial dysfunction characteristic of MNGIE.

### 4.6 Genetic Contribution: A Homozygous R44P Variant in the Chinese Population

The variant identified in our proband (c.131G>C, p.R44P) is absent from population databases (gnomAD) and has not been cataloged in HGMD. Based on ACMG guidelines, we classified it as likely pathogenic (Class 4), meeting the following criteria: PM2 (absent from gnomAD), PM3 (same residue previously implicated in MNGIE), PP3 (multiple in silico predictions support pathogenicity), and PP4 (patient phenotype highly specific for MNGIE). We acknowledge that functional and segregation data are lacking, which precludes a definitive “pathogenic” classification.

Notably, Xu et al. recently reported a Chinese patient with compound heterozygous *TYMP* variants (c.131G>C, p.R44P and c.1268 T>G, p.L423R) (Xu et al. 2025), indicating that R44P occurs in the Chinese population. In contrast to Xu's patient—who carried R44P in a compound heterozygous state—our proband harbors the same variant in a homozygous state. This genetic heterogeneity at the same residue highlights the complexity of TYMP‐related MNGIE and reinforces the critical role of codon 44 in TP function. Together, our findings and those of Xu et al. provide complementary evidence suggesting that R44P may be sufficient to disrupt TP function and drive disease pathogenesis.

### 4.7 Limitations

Several limitations should be acknowledged. First, functional validation—such as measurement of TP enzyme activity or plasma thymidine/deoxyuridine levels—was not performed. These biochemical assays would provide definitive evidence of pathogenicity but were not feasible due to the retrospective nature of the study and the lack of available blood samples. Second, parental DNA samples were unavailable for segregation analysis. Therefore, the variant is presented as “likely pathogenic” pending future functional confirmation. Third, our structural and docking analyses are computational predictions and should be interpreted as hypothesis‐generating rather than definitive.

## 5 Conclusions

Through integrated genomic and structural analyses, we propose the *TYMP* variant (c.131G>C, p.R44P) as a strong candidate likely pathogenic variant underlying MNGIE. Our computational modeling suggests that this substitution is likely to destabilize the TP quaternary structure, thereby potentially impairing both its dimerization and catalytic function. These predicted perturbations are consistent with a disease mechanism driven by thymidine accumulation and subsequent mitochondrial genomic instability.

Collectively, our findings expand the variational spectrum of *TYMP* and highlight the clinical heterogeneity of MNGIE, reinforcing the importance of considering this disorder in patients with unexplained multisystem manifestations. The identification of a second pathogenic substitution at codon 44 reinforces the critical role of this residue in TP function. Future functional studies are warranted to elucidate the precise mechanisms by which codon 44 pathogenic variants disrupt TP function and to validate the structural predictions presented here.

## Author Contributions

Songhua Zhao and Mingyi Ma conceived the project and designed the study. Ling Li, Xiu Chen, and Hua Li enrolled the cohort and collected the clinical data. Songhua Zhao conducted bioinformatic analysis. Kai Guo and Ting Zhang analyzed the data. Songhua Zhao wrote the manuscript. All authors revised the manuscript and approved the final version.

## Funding

This work was supported by the Southwest Medical University (00040164 and 2025SKLYB04) and Sichuan Science and Techology Program (2024NSFSC1641).

## Ethics Statement

Informed consent was obtained from all participating subjects. The research protocol was conducted in accordance with the Declaration of Helsinki guidelines and approved by the Ethics Committee of the affiliated hospital of Southwest Medical University (Approval No. KY2023410).

## Conflicts of Interest

The authors declare no conflicts of interest.

## Supporting information


**Data S1:** Laboratory findings.

## Data Availability

The data that supports the findings of this study are available in the Supporting Information of this article.
