# Supplementary material for: Association of a Homozygous TYMP c.131G>C Variant With MNGIE in a Chinese Pedigree: Insights From Genetic Analysis and Computational Modeling
Source: Mol Genet Genomic Med. 2026 Jun 17;14(6):e70253. doi: 10.1002/mgg3.70253 (PMC13275529; doi:10.1002/mgg3.70253)
Supplement: Supplementary file 1 — Data S1: Laboratory findings. [file MGG3-14-e70253-s001.docx]

Laboratory Findings

**Complete Blood Count:**

WBC: 12.06 × 10⁹/L (ref. 3.5–9.5) ↑,

NEU: 9.97 × 10⁹/L (ref. 1.8–6.3) ↑,

NEU-R: 82.70% (ref. 40–75) ↑,

RBC: 3.93 × 10¹²/L (ref. 4.3–5.8) ↓,

HGB: 123 g/L (ref. 130–175) ↓.

**Liver Function (I):**

ALB: 39.4 g/L (ref. 40–55) ↓,

DBIL: 8.0 μmol/L (ref. 0–7) ↑,

TBA: 11.6 μmol/L (ref. 0–10) ↑.

**Renal Function (Serum):**

Crea: 53.8 μmol/L (ref. 57–97) ↓.

**Electrolytes:**

Ca: 1.88 mmol/L (ref. 2.11–2.52) ↓,

**Coagulation (DIC Panel):**

PT: 13.5 s (ref. 9.8–12.1) ↑,

Fib: 4.68 g/L (ref. 1.8–3.5) ↑.

**Urinalysis:**

RBC: 12/μL (ref. 0–5) ↑.

**Lipid Profile:**

ApoB: 0.67 g/L (ref. 0.8–1.55) ↓.

**Thyroid Function:**

TgAb: 155.28 IU/mL (ref. 0.00–120.00) ↑.

**Tumor Markers (Serum, Male Panel A):**

TG: 0.73 ng/mL (ref. 3.80–77.00) ↓.

FER: 381.04 ng/mL (ref. 25.00–280.00) ↑,

CA724: 32.70 IU/mL (ref. 0.00–6.00) ↑,

SCCA: 7.29 ng/mL (ref. 0.00–2.50) ↑,

ProGRP: 73.66 pg/mL (ref. 0.00–67.42) ↑.

Procalcitonin (PCT): 0.061 ng/mL (ref. 0–0.05) ↑.
